# A cohort study of 4,190 patients treated with low-intensity pulsed ultrasound (LIPUS): findings in the elderly versus all patients

**DOI:** 10.1186/s12891-015-0498-1

**Published:** 2015-03-01

**Authors:** Robert Zura, Samir Mehta, Gregory J Della Rocca, John Jones, R Grant Steen

**Affiliations:** Department of Orthopaedic Surgery, Duke University Medical Center, Durham, NC USA; Department of Orthopaedic Surgery, Hospital of the University of Pennsylvania, Philadelphia, PA USA; Department of Orthopaedic Surgery, University of Missouri, Columbia, MO USA; Medical Affairs, Bioventus LLC, Durham, NC USA

**Keywords:** Nonunion fracture, Age, Obesity, Smoking, Diabetes, Osteoporosis, Arthritis, NSAIDs

## Abstract

**Background:**

Patient age is one of many potential risk factors for fracture nonunion. Our hypothesis is that older patients (≥60) with fracture risk factors treated with low-intensity pulsed ultrasound (LIPUS) have similar heal rate (HR) to the population as a whole. We evaluate the impact of age in conjunction with other risk factors on HR in LIPUS-treated patients with fresh fracture (≤90 days old).

**Methods:**

The Exogen Bone Healing System is a LIPUS device approved in 1994 to accelerate healing of fresh fracture. After approval, the FDA required a Post-Market Registry to assess performance. Patient data collected from October 1994 until October 1998 were individually reviewed and validated by a registered nurse. Four distinct data elements were required to report a patient: date fracture occurred; date treatment began; date treatment ended; and a dichotomous outcome of healed v. failed, by clinical and radiological criteria. Data were used to calculate two derived variables; days to treatment (DTT) and days on treatment (DOT). Every validated fresh fracture patient with DTT, DOT, and outcome is reported.

**Results:**

The validated registry had 5,765 patients with fresh fracture; 73% (N = 4,190) are reported, while 13% of patients were lost to follow-up, 11% withdrew or were non-compliant, and 3% died or are missing outcome. Among treatment-compliant patients, HR was 96.2%. Logistic estimates of the odds ratio for healing are equivalent for patients age 30 to 79 years and all age cohorts had a HR > 94%. Open fracture, current smoking, diabetes, vascular insufficiency, osteoporosis, cancer, rheumatoid arthritis, and prescription NSAIDs all reduced HR, but older patients (≥60) had similar HRs to the population as a whole. DTT was significantly shorter for patients who healed (p < 0.0001).

**Conclusions:**

Comorbid conditions in conjunction with aging can reduce fracture HR. Patients with fracture who used LIPUS had a 96% HR, whereas the expected HR averages 93%. Time to treatment was significantly shorter among patients who healed (p < 0.0001), suggesting that it is beneficial to begin LIPUS treatment early. Older patients (≥60) with fracture risk factors treated with LIPUS exhibit similar heal rates to the population as a whole.

## Background

Risk factors for fracture nonunion include patient age and the medical comorbidities attendant to age, as well as characteristics of the fracture itself [1]. The relationship of patient age to fracture heal rate (HR) was clearly demonstrated in a prospective study of 1,133 patients with intracapsular fracture of the femoral neck treated with internal fixation [2].

Few studies specifically address patient age as a risk factor for nonunion. Nevertheless, every study that has a broad inclusion range for age potentially has age as a confounder of HR. While age may be a risk in itself, it is also correlated with many known or suspected risk factors for nonunion [1]. For example, obesity increases fracture risk even in young women [3]; yet body-mass index (BMI) tends to increase with age [4] and with a range of comorbid illnesses that also increase with age, including diabetes, metabolic syndrome, insulin resistance, and inflammation [5]. Cigarette smoking is a risk for fracture [6] and for fracture nonunion [7] and smoking risks are dose-dependent and greatest among the elderly [8]. Similarly, osteoporosis risk and fracture risk increase with age [9], though young women with fracture can have bone microarchitecture characteristic of patients with osteoporosis [10]. Hence, to evaluate the effect of patient age specifically on HR, it may be necessary to assess many patients.

We report on the impact of age on HR among patients with fresh fracture, defined as fracture ≤90 days old. We used a 90-day cut-off because the Department of Health and Human Services defined long bone nonunion as occurring “when serial radiographs have confirmed that fracture healing has ceased for three or more months prior to starting treatment” [11]. Data are drawn from a post-market registry of the Exogen device (Bioventus LLC, Durham, NC), which has been published in part previously [12,13]. This study reports on the full, validated data set, including twice the number of patients reported previously. Our hypothesis is that older patients (≥60) with fracture risk factors treated with low-intensity pulsed ultrasound (LIPUS) have similar HR to the population as a whole.

## Methods

The Exogen Bone Healing System was approved by the FDA in 1994 for accelerating the time-to-heal of fresh fractures. The FDA required that the Exogen Post-Market Registry be open for at least 2 years, beginning in 1994 (study protocol available upon request). The registry was set up and maintained by a third-party consultant (Enterprise Software Solutions (ESS), Charlotte, NC). The present study was designed as a single arm, observational study of a convenience sample of consecutive consenting patients who had enrolled prospectively in the registry, and data were gathered and analyzed in a blinded fashion.

This study was exempted from ethical approval by the Institutional Review Board of Duke University Medical Center because data were drawn from a registry meant to satisfy U.S. Food and Drug Administration (FDA) requirements. Patients signed an informed consent at registry enrollment.

Data for the period from 14 Oct 1994 until 15 Oct 1998 were validated by a registered nurse who went through every patient record manually and compared the paper record to the digital record.

Inclusion criteria for the present analysis required four distinct data elements for each patient:

- **Date of fracture**: Calendar date when the fracture occurred.
- **Date of LIPUS treatment**: Calendar date when LIPUS treatment began.
- **Date that LIPUS treatment ended**: Calendar date when LIPUS treatment ended.
- **Outcome**: A dichotomous variable of healed or not healed at treatment end. For a fracture to be considered healed, the registry protocol specified that a fracture had to meet both clinical and radiological criteria:
  - ○ No motion or crepitus at the fracture site and free of pain on manual stress.
  - ○ At least three of four cortices bridged on X-ray views.

These data were used to calculate 2 derived variables of interest, in addition to Outcome:

- **Days-to-treatment (DTT)**: Time from fracture to beginning of LIPUS treatment. DTT defined the cohort of patients described here (DTT ≤ 90 days).
- **Days-on-treatment (DOT)**: Time the patient used LIPUS before attaining an outcome.

Additional patient data were analyzed for up to 181 different variables including:

- Patient demographics (*e.g*., age, sex, weight, height, body-mass index (BMI), etc.);
- Bone fractured (*e.g*., clavicle, femur, humerus, radius, scaphoid, tibia, ulna, etc.);
- Type of fracture (*e.g*., simple, closed, comminuted, stress, displaced, open, etc.);
- Type of treatment (*e.g*., cast, screws, nails, plates, external fixation, etc.).
- Medical comorbidities (*e.g*., smoking history, physician diagnosis of diabetes, cancer, hypertension, cardiovascular disease, osteoporosis, renal failure, etc.);
- Use of medications (*e.g*., analgesics, anticoagulants, antidiabetics, bisphosphonates, calcium channel blockers, diuretics, other cardiac medications, NSAIDs, steroids, etc.).

The Exogen device has an integrated treatment counter which counts both treatment episodes and treatment duration; this counter was downloaded to determine whether the patient was compliant with treatment. Every compliant patient in the manually-validated database with fresh fracture (≤90 days old) was included in this analysis, if DTT, DOT, and outcome data were available. To address whether there was systematic loss to follow-up in a specific risk group, patients with DTT, DOT, but no treatment outcome, were contrasted with patients with all necessary information. Patients with delayed union or fracture nonunion (90–365 days old) or with treatment-resistant fracture nonunion (>365 days old) were not included in this analysis. Because patients were drawn from a registry, no untreated control patients were available for comparison.

The t-statistic (Satterthwaite method for unequal variances) was used to compare means and Fisher’s exact test was used to test for association in 2×2 tables. P-values < 0.01 are reported. We calculate 95% confidence intervals (CIs) for percent healed point estimates. All data were analyzed using SAS software, v9.3 (Cary, NC).

## Results

The number of patients with complete records in the validated registry is 7,884 (Table 1), of whom 4,190 patients had fresh fracture, defined as a fracture ≤90 days old. Overall, 72.7% of all fresh fracture patients who received LIPUS treatment are reported (=4,190/5,765), while 12.8% of patients were lost to follow-up, 5.8% of patients were deemed non-compliant, 5.3% of patients withdrew from treatment, and 3.4% died or were missing an outcome. We do not report HR on an intention-to-treat (ITT) basis, because patient compliance is generally low outside the context of a clinical trial [14]; the HR we report is essentially efficacy with patient compliance. Among 7,884 compliant registry patients, HR was 93.9%. In the 4,190 patients with fresh fracture, the HR was 96.2% overall (Table 1).

**Table 1:** **Summary of the disposition of fracture records in the Exogen Registry database**

	**All fractures**	**0-90 days**	**91-365 days**	**>365 days**
**Any record in the Registry**	11,433	5,765	4,382	1,286
Deceased	42	25	14	3
Lost to follow-up	1,556	740	609	207
Non-compliant	776	333	330	113
Withdrew	691	304	286	101
Other	37	14	13	10
Missing treatment outcome	447	159	203	85
**Analyzed records in the Registry**	7,884	4,190	2,927	767
Healed	7,402 (93.9%)	4,032 (96.2%)	2,709 (92.6%)	661 (86.2%)
Failed	482 (6.1%)	158 (3.8%)	218 (7.4%)	106 (13.8%)

Males comprised 58.4% of all patients, and the average patient age was 43.3 years (Table 2). Patients lost to follow-up were significantly younger than patients with a treatment outcome (p < 0.0004), were somewhat heavier (p < 0.002), and had smoked for a longer period of time (p < 0.0002), but did not differ demographically in other ways (Table 3).

**Table 2:** **Summary of patient demographic data**

**Variable**	**Mean**	**SD**	**N**	**Median**
Age (years)	43.3	18.2	3,906	42.0
Weight (pounds)	171.0	43.3	3,108	170.0
Height (inches)	67.9	4.7	3,120	68.0
Body-Mass Index	25.9	5.5	3,092	25.1

This table includes 2,298 men (58.4% of the sample) and 1,639 women (41.6% of the sample) with fresh fracture.

**Table 3:** **Comparison of patients with an outcome in the registry to patients lacking an outcome**

	**Outcome (± SD)**	**N or %**	**No outcome (± SD)**	**N or %**	**Significance**
Patient age (years)	43.3 (±18.1)	4,157	41.4 (±17.9)	1,539	0.0004
Weight (lb)	170.5 (±43.0)	3,296	176.5 (±55.8)	1,030	0.002
Height (in)	67.9 (±4.7)	3,309	68.4 (±4.4)	1,031	0.0004
Body-mass index	25.9 (±5.5)	3,278	26.4 (±7.8)	1,021	NS
Days-to-treatment (mean)	38.6 (±24.5)	4,170	39.6 (±25.3)	1,567	NS
Days-on-treatment (mean)	118.5 (±86.0)	4,190	116.4 (±107.0)	1,561	NS
Female (vs. male) (%)**	1,745 (vs. 2,441)	41.7%	599 (vs. 957)	38.5%	NS
Open (vs. closed fracture) (%)**	710 (vs. 3,320)	17.6%	243 (vs. 1,245)	16.3%	NS
Number of prior procedures (mean)	1.5 (±0.8)	991	1.4 (±0.7)	368	NS
Number of comorbidities (mean)	1.4 (±0.7)	763	1.5 (±0.9)	250	NS
Number of medications (mean)	0.4 (±0.7)	2,747	0.4 (±0.6)	755	NS
Smoking years (mean)	5.1 (±10.6)	3,183	6.6 (±11.3)	1,041	0.0002

This approach should be very sensitive to risk factors that increase the risk of loss to follow-up. The p values shown are from T-tests, except for comparisons with an asterisk (**), which were tested using Fischer’s exact test because they are dichotomous variables.

**Figure 1:**
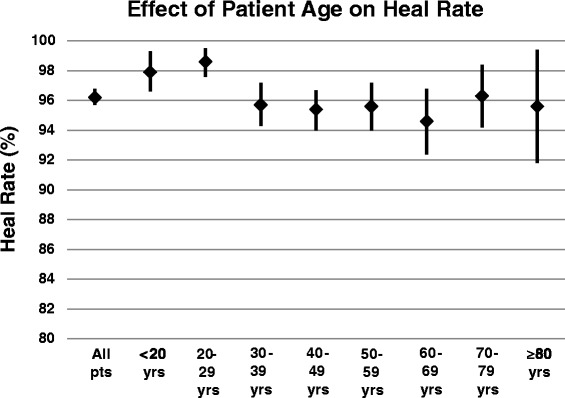
**Summary of the effect of patient age on heal rate (%).** The heal rate (HR) for each decade in shown, together with the upper and lower 95% confidence interval (CI) for each HR estimate. The HR is significantly higher than the overall HR only in patients age 20–29 years of age (p < 0.003); the HR did not differ significantly by age among patients older than age 30.

Our data show clearly that age did not have any effect on fracture HR, among patients age 30 or older (Figure 1). For patients 20–29 years old, HR was significantly higher than in older patients (p < 0.003). Logistic estimates of the odds ratio for healing are equivalent for patients age 30 to 79 years, and the HR for 298 LIPUS-treated patients age 70 to 79 years was nearly identical to the overall HR (Figure 1). All LIPUS-treated age groups had a HR > 94% (Figure 1).

**Figure 2:**
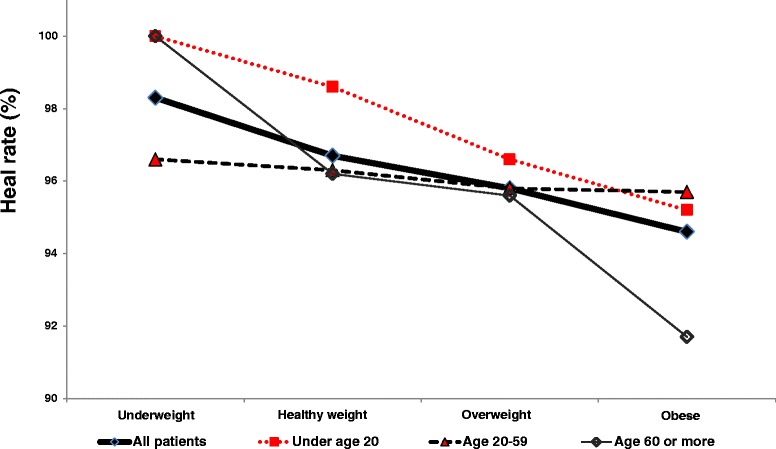
**Effect of age and BMI on heal rate (HR).** HR shows a decreasing trend with increasing weight, but HR tends to cluster by age, except in obese patients more than 60 years old. This suggests that BMI generally has more impact on HR than does patient age.

The relationship between patient age and fracture HR may be confounded by certain risk factors that correlate with age. For example, body-mass index (BMI) interacts with patient age in a complex manner. Patients who are obese generally do not heal as well as people of ideal weight (Figure 2). Obese people older than age 60 have a lower HR than either young obese people or elderly (≥60 years of age) people who are not obese. Patients under age 20 years, and underweight patients, have the highest HR (Figure 2).

The HR with LIPUS was evaluated for each bone studied (Table 4). In the overall sample, open fractures, as well as fractures of the tibia/fibula, femur, humerus, clavicle, radius/ulna, and metacarpal had significantly lower HR than the average of all bones, even when treated with LIPUS. Fractures of the metatarsal, radius, scaphoid, ankle, fibula, and ulna had significantly better HR than average when treated with LIPUS. Bones of elderly patients (>60 years old) had a similar HR to all patients, when treated with LIPUS.

**Table 4:** **Summary of heal rate by bone**

	**Overall**		**Heal**			**Elderly**		**Heal**
**Comorbidity**	**Healed**	**Failed**	**Rate**	**Lower CI**	**Upper CI**	**Healed**	**Failed**	**Rate**
All fractures	4,032	158	96.2%	95.7%	96.8%	780	38	*95.4%*
All closed fractures	3,212	108	96.7%	96.1%	97.4%	650	27	*96.0%*
All open fractures	147	9	*94.2%*	90.6%	97.9%	24	0	**100.0%**
Tibia	939	42	95.7%	94.5%	97.0%	170	9	95.0%
Tibia/Fibula	705	33	*95.5%*	94.0%	97.0%	140	7	95.2%
Femur	412	19	*95.6%*	93.7%	97.5%	136	4	97.1%
Metatarsal	423	7	**98.4%**	97.2%	99.6%	40	0	**100.0%**
Radius	337	2	**99.4%**	98.6%	100.0%	94	1	98.9%
Humerus	207	19	*91.6%*	88.0%	95.2%	60	11	*84.5%*
Scaphoid	203	6	**97.1%**	94.9%	99.4%	11	0	**100.0%**
Clavicle	120	7	*94.5%*	90.5%	98.5%	7	1	*87.5%*
Ankle	122	3	**97.6%**	94.9%	100.0%	31	2	*93.9%*
Radius/Ulna	144	7	*95.4%*	92.0%	98.7%	36	0	**100.0%**
Fibula	96	2	**98.0%**	95.2%	100.0%	7	0	100.0%
Ulna	84	2	**97.7%**	94.5%	100.0%	14	0	100.0%
Metacarpal	42	2	*95.5%*	89.3%	100.0%	4	0	100.0%

Every bone represented in the database by more than 50 fractures is tabulated. Column totals do not always add up because some information is missing; for example, some fractures were not defined as either open or closed. The total of all bones is larger than the total count of patients because some patients broke multiple bones. The heal rate (HR) in humerus is significantly lower than the overall HR because the confidence interval (CI) for humerus does not overlap the CI for “All fractures.” **Bolded HR numbers** are above the CI associated with “All fractures.” *Italic HR numbers* are below the CI associated with “All fractures.” In the “Elderly” group, **bolded HR numbers** are above the CI for the corresponding fracture in the whole cohort, while *italic* HR numbers are below the CI for the corresponding fracture in the whole cohort. Elderly HR is comparable to the HR of the overall sample.

We compared patients who healed with LIPUS to patients who failed to heal (Table 5). Patients who failed to heal were on average 4.5 years older than patients who healed (p < 0.0009). Days-to-treatment (DTT) was significantly shorter for patients who healed (p < 0.0001); this strongly suggests that it is beneficial to begin treatment with LIPUS as soon as possible after fracture. Days-on-treatment (DOT) was also significantly shorter for patients who healed (p < 0.0001), but this would be expected, as patients who failed to heal would likely continue using the treatment for a longer time. Patients with open fracture were more at risk of treatment failure (p < 0.002). Patients who failed to heal were also likely to be using more medications (p < 0.003), although there was no significant difference in the number of medical comorbidities between these two groups (Table 5).

**Table 5:** **Comparison of fresh-fracture patients who healed with LIPUS to patients who did not heal**

	**Healed (± SD)**	**N or %**	**Failed (± SD)**	**N or %**	**Significance**
Patient age (years)	43.2 (±18.1)	4,000	47.7 (±16.7)	157	0.0009
Weight (lb)	170.4 (±43.1)	3,166	173.9 (±41.9)	130	NS
Height (in)	67.9 (±4.7)	3,181	67.0 (±4.4)	128	NS
Body-mass index	25.8 (±5.5)	3,150	26.9 (±5.8)	128	NS
Days-to-treatment (mean)	38.3 (±24.3)	4,013	47.1 (±27.3)	157	0.0001
Days-on-treatment (mean)	115.6 (±83.1)	4,032	193.0 (±119.7)	158	<0.0001
Female (vs. male) (%)**	1,674 (vs. 2,356)	41.5%	71 (vs. 85)	45.5%	NS
Open (vs. closed fracture) (%)**	669 (vs. 3,212)	17.2%	41 (vs. 108)	27.5%	0.002
Number of prior procedures (mean)	1.4 (±0.8)	950	1.6 (±1.2)	41	NS
Number of comorbidities (mean)	1.4 (±0.7)	727	1.4 (±0.5)	36	NS
Number of medications (mean)	0.4 (±0.7)	2,639	0.7 (±0.9)	108	0.003
Smoking years (mean)	5.0 (±10.6)	3,062	7.2 (±11.8)	121	NS

This approach should be very sensitive to risk factors that increase the risk of treatment failure. The p values shown are from T-tests, except for comparisons with an asterisk (**), which were tested using Fischer’s exact test because they are dichotomous variables.

Elderly patients with comorbidities had a HR comparable to the overall patient sample (Table 6). Patients with diagnosed cardiovascular or renal disease had a HR better than average. But patients who smoked or had diabetes, vascular insufficiency, osteoporosis, cancer, or rheumatoid arthritis had a lower HR than average. Nevertheless, there was not a clinically significant difference in HR as a function of comorbidity among LIPUS-treated patients, and elderly patients had a HR comparable to the overall sample (Table 6).

**Table 6:** **Impact of comorbidity on heal rate (HR) in the fresh fracture cohort**

	**Overall**		**Heal**			**Elderly**		**Heal**
**Comorbidity**	**Healed**	**Failed**	**Rate**	**Lower CI**	**Upper CI**	**Healed**	**Failed**	**Rate**
All fractures	4,032	158	96.2%	95.7%	96.8%	554	28	*95.2%*
Current smokers	652	35	*94.9%*	93.3%	96.5%	81	1	**98.8%**
Diabetes	224	11	*95.3%*	92.6%	98.0%	110	4	96.5%
Hypertension	188	7	96.3%	93.6%	99.0%	83	2	97.6%
Vascular insufficiency	107	5	*95.5%*	91.7%	99.4%	56	2	96.6%
Osteoporosis	80	5	*94.1%*	89.1%	99.1%	57	5	91.9%
Cancer	77	4	*95.1%*	90.3%	99.8%	50	2	96.2%
Cardiovascular disease	60	2	**97.2%**	93.3%	100.0%	54	2	96.4%
Alcoholism	68	3	95.8%	91.1%	100.0%	10	0	100.0%
Renal disease	44	1	**97.8%**	93.5%	100.0%	11	1	*91.7%*
Rheumatoid arthritis	36	4	*90.0%*	80.7%	99.3%	21	3	87.5%

The overall heal rate (HR) includes all patients, even if they have comorbidities or are older than age 60. Then various comorbidities are broken out, for the entire fresh fracture cohort overall, and for the elderly cohort (≥60 years of age) specifically. **Bolded HR numbers** are above the CI associated with “All fractures.” *Italic HR numbers* are below the CI associated with “All fractures.” In the “Elderly” group, **bolded HR numbers** are above the CI for the corresponding fracture in the whole cohort, while *italic* HR numbers are below the CI for the corresponding fracture in the whole cohort. Elderly HR is comparable to the HR of the overall sample.

Patients taking analgesics, prescription NSAIDs, anticoagulants, steroids, antibiotics, insulin, or calcium channel blockers were impaired in HR, compared to the overall cohort of patients (Table 7). However, patients taking non-prescription NSAIDs were not impaired in healing.

**Table 7:** **Impact of current medication use on heal rate (HR) in the fresh fracture cohort**

	**Overall**		**Heal**			**Elderly**		**Heal**
**Medication**	**Healed**	**Failed**	**Rate**	**Lower CI**	**Upper CI**	**Healed**	**Failed**	**Rate**
No medications at all	1,765	61	96.7%	95.8%	97.5%	228	11	*95.4%*
Non-NSAID analgesic	301	19	*94.1%*	91.5%	96.7%	48	4	92.3%
Prescription NSAIDs	159	14	*91.9%*	87.8%	96.0%	40	4	90.9%
Anticoagulants	107	9	*92.2%*	87.4%	97.1%	54	2	96.4%
Non-prescription NSAIDs	104	3	97.2%	94.1%	100.0%	31	0	100.0%
Steroids	93	8	*92.1%*	86.8%	97.3%	30	5	*85.7%*
Antibiotics	80	4	*95.2%*	90.7%	99.8%	14	2	*87.5%*
Insulin	75	6	*92.6%*	86.9%	98.3%	36	2	94.7%
Calcium channel blockers	62	6	*91.2%*	84.4%	97.9%	37	3	92.5%

The overall average includes all patients, even if they use medications or are older than age 60. Then various medications are broken out, for the entire fresh fracture cohort overall, and for the elderly cohort (≥60 years of age) specifically. The HR for each comorbidity in shown, together with the upper and lower 95% confidence interval (CI) for each HR estimate. *Italic HR numbers* are below the CI associated with “All fractures.” In the “Elderly” group, *italic* HR numbers are below the CI for the corresponding fracture in the whole cohort. Elderly HR was comparable to the HR of the overall sample.

## Discussion

Patients with fresh fracture who used LIPUS had a 96% HR (Table 1), whereas the HR from the literature averages 93% [15-20]. Thus, LIPUS may have reduced the nonunion rate by up to 40%, with respect to that literature [15-20]. Age did not have a significant impact on fracture HR, and the HR for patients aged 30 to 79 years was equivalent (Figure 1). However, HR is confounded by other risk factors for nonunion, such as elevated BMI (Figure 2). Fracture HR varies as a function of the bone broken (Table 4), but the most compelling risks for treatment failure are patient age, open fracture, treatment delay, and use of medications (Table 5). HR can be reduced by medical comorbidities (Table 6) and by medications such as prescription NSAIDs, anticoagulants, steroids, and calcium channel blockers (Table 7). Unlike prescription NSAIDs, non-prescription NSAIDs do not have a significant effect on HR (Table 7).

Evaluation of LIPUS-treated patients over age 30 did not show the decline in HR (Figure 1) often described in the literature [15-20]. However, patients who failed to heal with LIPUS (Table 5) were significantly older than patients who did heal. We surmise that this apparent discrepancy is explained by LIPUS-treated patients under age 30, who healed well and reduced the average age of healed patients (Table 5).

There is strong evidence in the literature that increasing age is associated with impaired fracture healing [2,21,22]. Femoral neck HR decreased with age, both in displaced (p < 0.001) and non-displaced (p < 0.003) fractures [2]. Among patients under age 60, just 7 of 106 had fracture nonunion (HR = 93.4%); among patients age 70 to 80 years, 84 of 337 had nonunion (HR = 75.1%) [2]. In a separate study, patients with displaced femoral neck fracture who developed nonunion were significantly older than patients with normal healing [21]. Nevertheless, in a third study, age was not identified as a predictor of nonunion in 202 patients with intracapsular femoral neck fracture [22].

Patient age has an impact on tibial healing in the absence of LIPUS [23-26]. Among 42 patients treated surgically for tibial plateau fracture, the failure rate was just 7% in young patients, but increased to 79% in patients older than age 60 [23]. Age was a risk factor for total knee arthroplasty among 8,426 patients (p < 0.0001) with tibial plateau fracture [24]. Increasing patient age was associated with worse surgical outcomes after tibial surgery [25,26]. Yet age was not noted as a nonunion risk factor in studies of 211 patients with long bone fracture [20] or 416 patients with tibial shaft fracture [27].

Age can also have an impact on healing in humeral fracture. A retrospective review of nonunions of the midshaft humerus concluded that advanced age accounted for 57% of nonunions [28]. A prospective study of 110 elderly patients with humeral diaphyseal fracture found that nonunion was predicted by patient age (p < 0.05), prior stroke (p < 0.001), or prior nonunion (p < 0.001) [29]. In a review of 37 patients with humeral fracture, the nonunion rate was 10.8% and patient age was the only significant predictor of humeral nonunion [30]. Finally, age is a risk for nonunion in clavicular fracture. Among 337 patients with clavicular fracture, patient age was an important predictor of nonunion [31], and older patients with clavicular fracture typically had a higher risk of nonunion [32,33].

Our data suggest that patients who are obese generally do not heal as well as people of ideal weight (Figure 2). Obesity has been recognized as a risk factor for fracture nonunion for decades [34], and BMI is significantly higher in patients with failed reduction of the distal tibia [35]. Obesity (BMI ≥30) is also a significant risk factor (p < 0.01) for required revision of total hip replacement after femoral neck fracture [36], and BMI is known to interact with risk factors for nonunion, including smoking [37].

A major strength of the registry database is the size of the cohort, which we believe to be the largest ever reported. This enabled us to gain insight into complex relationships. For example, BMI interacts with patient age in a complex way (Figure 2); obese patients generally do not heal as well as non-obese patients. Patients older than age 60 who are obese have a substantially lower HR than either young obese patients or elderly (≥60 years of age) patients of ideal weight. This could be because patients who are elderly and obese can have a range of other health problems, including diabetes, hypertension, and vascular insufficiency (Table 6), and may be taking various medications (Table 7). Conversely, patients under age 20, or underweight patients, have a higher HR than other patient groups (Figure 2).

A second strength of this study is that patients enrolled in the registry did not differ in obvious ways from a random sample of fracture patients. Roughly 16.2% of registry patients were smokers in 1994 to 1998 (Table 6), which is consistent with the 18.1% of people who are currently smokers in the United States [38]. Roughly 5.6% of registry patients had diabetes, which is comparable to the 6.6% of Americans age 45 to 64 years who had diabetes in 1996 [39], the median year in the registry. Roughly 4.7% of registry patients had hypertension, somewhat less than the 10.5% of Americans (age 18–44 years) who had a diagnosis of hypertension between 2005 and 2008 [40].

A limitation of this study is that blinding was impossible, and patients may have been motivated to perceive a treatment benefit, even if none existed. Because the registry collated outcomes from treatment, it is a therapeutic study, and therapeutic studies are stronger, when blinded [41]. Results reported herein are a retrospective analysis of standardized, prospectively-collected treatment data, so they provide Level III evidence [41]. Yet the large number of patients (N = 4,190; see Table 1) and the relatively high rate of retention (73%; see Table 1), may overcome some of the limitations inherent to a registry study [42].

A second limitation of this study is that the data are as much as 20 years old. It has been argued that fracture management has not changed substantially since the registry opened [43]. Intramedullary nailing was accepted as treatment for long bone fracture prior to when the bulk of the registry patients were enrolled [44]. Therefore, many of the registry patients appropriate for nailing may have received that treatment. We cannot exclude the notion that changes in patient management may have had an impact on HR of patients in the registry, but we believe that such changes were probably evolutionary, not revolutionary.

A third limitation of our work is that registry studies typically capture a limited dataset based on clinical convenience, sacrificing data granularity for breadth of clinical capture. While it would be useful to have more detailed information about the precise location of each fracture within the bone, or the extent of associated soft tissue injury, or even why the physician elected to treat a fracture with LIPUS, these fields were not in the database.

A final limitation of this study is that the calculated number-needed-to-treat (NNT) for patients is currently 33, in order to avoid one nonunion. However, this calculation is misleading. If fresh fracture patients are selected for treatment based on the clinical understanding of risk factors contemporary with registry enrollment, then NNT would be 33. But a combination of advanced age and obesity decreases the HR substantially (Figure 2), and patients who are aged, obese, and diabetic may show a further reduction of HR. Therefore, the NNT for an aged, obese, and diabetic patient may be lower than 33, although a multivariate analysis will be required to test this hypothesis.

It is very important that fracture treatment in the elderly be effective, because there can be severe consequences of even brief physical inactivity. Sarcopenia is the loss of lean muscle mass that occurs even with exercise during healthy aging [45]. Elderly adults lose muscle mass and lean body tissue far more rapidly than do young adults during prolonged physical inactivity [46]. Ten days of experimental bed rest in otherwise-healthy 67-year-old adults resulted in a 14% loss of power, a 13% loss of strength, and a 12% loss of aerobic capacity [47]. Though physical performance was not impaired by bed rest among healthy adults [47], one might expect physical performance to be diminished in adults with fracture. Elderly patients hospitalized with acute illness experience a rapid functional decline [48]; among 71 patients (average age = 74 years), two-thirds experienced a functional decline by the second day in hospital. Assessments of physical mobility, toileting, incontinence, feeding, grooming, and mental status showed a continued decline during hospitalization in 10% of patients, and most patients had no improvement in functional ability until after release from the hospital [48]. A more recent study involving 2,293 patients (age ≥70) showed that one-third of patients declined in activities of daily living between hospitalization and discharge [49]. The frequency of functional decline increased markedly with age: 23% of patients aged 70–74 declined in function, but 63% of patients >90 years old experienced such a decline [49]. Femoral neck fracture in the elderly is associated with a 5-fold elevation in risk of mortality in 1 year, with deficits in mobility, respiratory and renal function, cognition, and endocrine function [50]. If LIPUS can be used to mitigate age as a risk factor for healing, then the severe impact of physical inactivity post-fracture can potentially be minimized.

## Conclusions

Our results suggest that older age can be mitigated as a risk factor for impaired fracture healing when LIPUS is used (Figure 1). Early use of LIPUS is associated with fracture healing in 96.2% of patients overall (Table 1) and in 95.2% of the elderly (Table 6). Medical comorbidities (*e.g*., smoking, diabetes, vascular insufficiency, osteoporosis, cancer, and rheumatoid arthritis; Table 6), and use of medications (*e.g*., prescription NSAIDs, anticoagulants, steroids, antibiotics, insulin, and calcium channel blockers; Table 7) may increase the risk of nonunion in the elderly. Yet elderly patients treated with LIPUS have a HR comparable to young patients (Figure 1) and delays in using LIPUS are associated with non-healing fracture (Table 5). Hence, we recommend that elderly patients with risk factors for nonunion be treated with LIPUS soon after injury.

## Abbreviations

BMI: Body-mass index
CI: Confidence interval
DOT: Days on treatment
DTT: Days to treatment
FDA: Food & Drug Administration
HR: Heal rate
LIPUS: Low-intensity pulsed ultrasound
N: Sample size
NNT: Number needed to treat
p: Probability
